# The Association between SOCS1−1656G>A Polymorphism, Insulin Resistance and Obesity in Nonalcoholic Fatty Liver Disease (NAFLD) Patients

**DOI:** 10.3390/jcm8111912

**Published:** 2019-11-08

**Authors:** Agnieszka Kempinska-Podhorodecka, Ewa Wunsch, Piotr Milkiewicz, Ewa Stachowska, Malgorzata Milkiewicz

**Affiliations:** 1 Department of Medical Biology, Pomeranian Medical University, 70-111 Szczecin, Poland; milkiewm@pum.edu.pl; 2 Translational Medicine Group, Pomeranian Medical University, 71-210 Szczecin, Poland; ewa.wunsch@gmail.com (E.W.); p.milkiewicz@wp.pl (P.M.); 3 Liver and Internal Medicine Unit, Medical University of Warsaw, 02-097 Warsaw, Poland; 4 Department of Human Nutrition and Metabolomics, Pomeranian Medical University, 71-460 Szczecin, Poland; ewa.stachowska@pum.edu.pl

**Keywords:** obesity, steatohepatitis, metabolic syndrome, cytokines

## Abstract

Suppressor of cytokine signaling (SOCS) proteins prevent uncontrolled cytokine signaling and appear to play a role in the pathological processes behind obesity and insulin resistance. The polymorphism of the *SOCS1* gene (rs243330, −1656G>A) is associated with obesity and glucose sensitivity. To estimate the effect of this *SOCS1* gene polymorphism on nonalcoholic fatty liver disease (NAFLD) susceptibility, we performed a study on 138 patients with ultrasound-confirmed NAFLD and 1000 healthy blood donors. The relationship between the SOCS1−1656G>A polymorphism and serum biochemical parameters in NAFLD was additionally investigated. The *SOCS1* variant was genotyped using a dedicated TaqMan assay. The frequency of rs243330 polymorphism did not differ between patients and controls. However, in a cohort of obese individuals (BMI ≥ 30 kg/m^2^) the occurrence of the G allele of the SOCS1−1656G>A polymorphism was strongly associated with NAFLD (odds ratio (OR) 1.6; 95% CI,1.1–2.5; *p* = 0.009), and carriers of the AA genotype have lower risk of developing NAFLD (OR 0.4; 95% CI, 0.2–0.7; *p* = 0.004). Overweight NAFLD patients who were carriers of GG genotypes had significantly lower levels of homeostasis model assessment of insulin resistance (HOMA-IR) values (*p* = 0.03 vs. AA), and the obese GG homozygotes had lower serum concertation of triglyceride (GG vs. AA; *p* = 0.02). Serum liver enzyme activities were not modified by the presence of *SOCS1* risk variants. In conclusion, the observed phenotype of overweight NAFLD patients with non-elevated levels of TG and HOMA-IR, which is associated with genetic variants of *SOCS1*, provides a rationale for further research on the pathophysiology of fatty liver disease.

## 1. Introduction

Nonalcoholic fatty liver disease (NAFLD) is an example of pathological fat accumulation in the liver. In the current obesity epidemic, NAFLD is among the most common causes of chronic liver disease [1]. This condition encompasses a spectrum of obesity-associated liver diseases, including nonalcoholic steatohepatitis (NASH), cirrhosis, and primary liver cancer [2]. The true prevalence of NAFLD and NASH is unknown, and several efforts have been made to identify patients with increased risk of progression to advanced fibrosis [3,4]. The exact proportion of patients who progress to cirrhosis is thought to be low (5%), but the rapidly escalating prevalence of obesity means that NASH has become one of the most common causes of chronic liver disease [1]. Unfortunately, NAFLD-associated cirrhosis is associated with complications that considerably add to patient morbidity and mortality, as well as to the cost for the healthcare system [5].

Little is known about the natural history of NAFLD. Disproportionate accumulation of triglycerides (TG) in hepatocytes is the hallmark of NAFLD, and the manifestation of steatosis is a major indicator of multiorgan insulin resistance, independent of body mass index (BMI), percent body fat, and visceral fat mass [1]. Dysregulated expression and/or secretion of transcription factors and cytokines, respectively, influence downstream metabolic pathways and play a crucial role in NAFLD pathogenesis. In obese individuals with NAFLD, the enhanced production of inflammatory cytokines—such as IL-6 and TNF-α—by adipose tissue macrophages may contribute to the development of insulin resistance [2]. Recently, emphasis has been placed on genetic factors that may have a role in NAFLD etiology [6], and some genetic polymorphisms involved in the development of the disease (e.g., PNPLA3, TM6SF2, APOB) were identified by a genome-wide association study (GWAS) [7]. To date, there is no approved pharmacological therapy for NAFLD, and the current treatment remains weight loss with lifestyle modifications and physical activity. 

Suppressor of cytokine signaling 1 (SOCS1) is one of the eight members of the SOCS family of regulatory proteins which are synthesized by early response genes in response to external stimuli, such as cytokines, growth factors, and toll-like receptors (TLRs) [8]. SOCS proteins play essential roles in mediating inflammatory responses in both immune cells and metabolic organs, such as the liver and adipose tissue [9], and they are involved in the pathogenesis of metabolic syndrome by modulation of either cytokine expression or insulin signaling. SOCS1 and SOCS3 act as negative regulators in insulin signaling and serve as one of the missing links between insulin resistance and cytokine signaling [10]. Hepatic expression of SOCS1 is elevated in rodent models of obesity and insulin resistance [11], and adenoviral-mediated *SOCS1* gene transfer into the mouse liver resulted in glucose intolerance and insulin resistance [12]. SOCS1 inhibits the production of proinflammatory cytokines and the granulocyte-macrophage colony-stimulating factor (GM-CSF) [13] as an inhibitor of Janus kinase/signal transducers and activators of transcription (JAK/STAT) signaling [14,15]. via Through inhibition of the JAK-STAT pathway, SOCS-activated TLR ligands inhibit type-1 interferons (IFNs) which, in conjunction with interleukin (IL-1) or tumor necrosis factor-α (TNF-α), trigger β-cell dysfunction and/or death [16].

On the other hand, there is a growing body of evidence supporting the role of SOCS proteins in the modulation of other types of signaling pathways; among these are insulin and insulin-like growth factor signaling [17]. Cytokine-induced SOCS1 interacts with the phosphorylated insulin receptor, thereby preventing binding and activation of insulin receptor substrates (IRS), which leads to the inhibition of insulin signaling and the induction of insulin resistance. Moreover, inflammation- induced expression of SOCS1 promotes IRS2 ubiquitination, and thus triggers insulin resistance [12]. A reduced level of IRS phosphorylation was observed both in animal models of type 2 diabetes and in type 2 diabetic patients [18]. Of note, the mutation in the promoter region of the *SOCS1* gene (rs243330, −1656G>A) was associated with obesity and insulin resistance in the general population [10,19].

Recently, SOCS1 has been shown to be implicated in the pathophysiology of the liver as SOCS1 expression is induced in hepatocytes by a variety of exogenous stimulations, such as IL-6 and IFN-γ [8]. In primary biliary cholangitis (PBC), hepatic expression of SOCS1 is inhibited by microRNA-155, which may result in the increased production of proinflammatory cytokines such as tumor necrosis factor α (TNFα) and interleukin 1β (IL-1β) [20]. Polymorphism of the promoter region of the *SOCS1* gene, the −1656G>A variant, was demonstrated as a risk factor for PBC, an autoimmune cholestatic liver disease [21].

Given that SOCS1 is often dysregulated in a wide variety of liver diseases, and that reduced insulin sensitivity and higher body mass index (BMI) are associated with genetic variants in the *SOCS1* gene, we examined the polymorphism in the promoter region of the *SOCS1* gene (position −1656; rs243330) for association with obesity and NAFLD-related serum quantitative traits. 

## 2. Materials and Methods

### 2.1. Patients and Controls

One hundred and thirty-eight patients (86 male and 52 female, median age 48 years, range 18–74 years) with features of NAFLD on a high-resolution B-mode abdominal ultrasound scanner (Acuson X300 Simens, San Jose, CA, USA) and scoring more than 2.0 in Hamaguchi score [22] for liver steatosis were included in the study (Table 1). The degree of fatty liver disease was assessed by a trained physician. Briefly, this score takes into account three major features detectable in ultrasound. These include (i) bright liver and hepatorenal echo contrast; (ii) deep attenuation; (iii) vessel blurring. We have used this score in our previous studies which confirmed its reliability and reproducibility in patients with NAFLD [23]. The examining physician was blinded to clinical and laboratory data of patients. A cohort of 1000 Caucasian (500 females and 500 males, median age 24 years, range 18–66 years) blood donors from the Regional Blood Donor Center in Szczecin (Poland) served as a control group (Table 1). They all had a medical checkup, and a good state of health was a prerequisite to qualify for blood donation. By definition, they need to have normal transaminases, gamma-glutamyltransferase (GGT), and glucose to become blood donors. Exclusion criteria in the studied group included alcohol consumption above the safe limit (< 20 g/day), pregnancy, and known cases of chronic viral hepatitis (C or B). All subjects were explained the purpose and nature of the study. Written and informed consent were obtained. All patients were Polish Caucasians living in the West Pomerania region of Poland. The study protocol conforms to the ethical guidelines of the 1975 Declaration of Helsinki (6th revision, 2008) and was approved by the Ethics Committee of Pomeranian Medical University (IRB no. KB-0012/57/11, and KB-0012/09/10).

A 10 mL sample of venous blood drawn after a 12 h overnight fast was obtained from each subject. In the group of NAFLD patients, plasma glucose, insulin, cholesterol, TG, and liver profile including aspartate aminotransferase (AST), alanine aminotransferase (ALT), and GGT were measured. Insulin resistance (IR) was assessed by using the readily accessible homeostasis model assessment of insulin resistance (HOMA-IR). Routine anthropometric measurements served as a basis for calculating BMI, total mass of fat in kilograms (F (kg)), and active components of fat-free mass in kilograms (TA (kg)). The range of BMI values was used according to WHO 1995 recommendations. Adipose tissue distribution was assessed using the waist-to-hip ratio (WHR).

### 2.2. Genotyping 

Genomic DNA was isolated from EDTA anticoagulated blood using the DNeasy Blood and Tissue Kit (Qiagen, Hilden, Germany). Genotyping of the SOCS1−1656G>A polymorphism (rs243330) was performed using TaqMan™ Gene Expression Assays (Assay ID: C_3189843_10, Applied Biosystems, Foster City, CA, USA). PCR reactions contained 20 ng DNA, 900 nM of each primer, 1× TaqMan Universal Master Mix, and 200 nM of VIC-labeled and FAM-labeled probes in 25 μL reactions. Amplification conditions were as follows: 95 °C for 10 min; 40 cycles of 92 °C for 15 s and 60 °C for 1 min. Fluorescence data were analyzed with allelic discrimination 7500 Software v.2.0.2 (Applied Biosystems, Foster City, CA, USA). Valid genotypic data were obtained for 100% of cases and controls. 

### 2.3. Statistical Analysis

All statistical analyses were performed using StatView version 5 software (SAS Institute Inc., Carry, NC, USA). The differences in allele frequency and genotype distribution between case and control subjects were compared by a chi-square test of association (Pearson) and odds ratios (ORs) with 95% confidence intervals (95% CI) were calculated. Associations between genotyped variants and tested variables were analyzed using nonparametric Mann–Whitney test or with ANOVA test, as appropriate. Data were presented as medians (and ranges) for continuous variables, and *p*-values less than 0.05 were considered to be statistically significant. The genotype frequencies of the *SOCS1* polymorphism were tested for consistency with Hardy–Weinberg equilibrium (HWE) using exact tests.

## 3. Results

The frequencies of the *SOCS1* alleles and genotypes among patients with NAFLD in comparison to controls are presented either for the whole cohort of subjects in each group (Table 2) or for the group of individuals with BMI ≥30 kg/m^2^ (Table 3). In the entire NAFLD cohort, there was a tendency toward higher frequency of the G allele (*p* = 0.08) and lower occurrence of AA genotype (*x*^2^ = 3.3; *p* = 0.07) in comparison to healthy controls (Table 2). A subsequent analysis of the obese subjects with BMI ≥ 30 kg/m^2^ showed a significantly higher frequency of G allele of SOCS1−1656G>A polymorphism in the group of NAFLD patients than for the obese controls (56.1% vs. 43.3%; OR = 1.6, *p* = 0.009 vs. controls), whereas the AA genotype was presented with lower frequency in obese NAFLD patients in comparison to obese controls (16.3% vs. 33.6%, *p* = 0.004; Table 3)

In a cohort of 1000 healthy Caucasians, we noticed a prevalence of obese individuals (BMI ≥ 30 kg/m^2^) among carriers of the AA genotype (33.6% obese vs. 24.7% normal weight, *p* = 0.03; Table 4). Because there was a small number (*n* = 16) of those who were underweight (BMI ≤ 18.5 kg/m^2^) they were excluded from the data analysis. We did not identify any departure from Hardy–Weinberg equilibrium (HWE) either in the whole cohort or in the separate analyses performed in males and females (all *p* > 0.05).

The SOCS1−1656G>A polymorphism was also analyzed for associations with serum biochemical parameters such as TG, AST, ALT, GGT, and HOMA-IR in NAFLD patients. In the entire cohort of NAFLD patients, no association was observed for the SOCS1−1656G>A polymorphism and the examined traits (Table 5). To investigate within-group differences, the group of NAFLD patients was stratified by BMI category and divided into three subgroups: obese (BMI > 29.9 kg/m^2^), overweight (BMI from 25 to 29.9 kg/m^2^), and lean (BMI < 25.0 kg/m^2^) patients. In our study group, there were only four lean NAFLD patients; therefore, in further analysis, we focused on the obese and overweight patients. Thus, within the group of overweight subjects with NAFLD, the homozygotes for the G allele had significantly lower levels of HOMA-IR in comparison to homozygotes for the A allele (1.8 ± 1.3 mg/dL in GG vs. 3.6 ± 2.2 mg/dL in AA, *p* = 0.03). Similarly, there was a tendency to lower levels of GGT in overweight GG homozygotes in contrast to AA homozygotes (*p* = 0.05; Table 6). In the group of obese NAFLD patients, the homozygous carriers of G allele had mean triglyceride levels in normal range and they were significantly lower than in AA homozygotes (mean 136.2 ± 54.6 mg/dL in GG vs. 181 ± 85.1 mg/dL in AA; *p* = 0.02).

## 4. Discussion

The results of this study, showing a significantly higher frequency of G allele and lower frequency of AA genotype of SOCS1−1656G>A polymorphism amongst overweight and obese NAFLD patients in comparison to weight-matched controls, suggest that G is a risk allele in metabolic liver diseases. Interestingly, in contrast to NAFLD in a healthy population, the AA genotype of this *SOCS1* variant predisposes carriers to obesity. Moreover, evidence for the association between the SOCS1−1656G>A polymorphism and triglyceride level or insulin resistance was shown in the group of overweight or obese NAFLD patients.

In our cohort of healthy subjects, we determined the frequency of *SOCS1* gene polymorphism and reported that GG (wild type) was present in 23.3%, GA in 52%, and AA in 24.7% of those with normal weight. The population of obese subjects was significantly enriched for the AA genotype, which accounted for 33.6% of that group (odds ratio = 1.5; *p* = 0.03 vs. normal weight). These results indicate that SOCS1−1656A variant is a risk factor for obesity. This is consistent with the study that reported a significant association of SOCS1−1656G>A polymorphism with obesity in young Danish Caucasians [19].

Generally, obese and overweight individuals have higher free fatty acid levels, which stimulate insulin resistance [24]. There is an association between insulin resistance and BMI-defined obesity [19,25,26], and the prevalence of NAFLD increases from 74% to 80% in obese individuals [5,27,28]. In the entire group of our NAFLD patients, there was a tendency to higher frequency of the G allele, and to lower frequency of homozygous AA in comparison to healthy controls (*p* = 0.08 and *p* = 0.07, respectively). This bias was even more pronounced amongst obese subjects and, thus, the prevalence of the G allele of the SOCS1−1656G>A polymorphism was significantly higher in obese NAFLD patients in comparison to obese control individuals (56.1% vs. 43.3%; *p* = 0.009), and in those patients, a significantly lower frequency of AA was observed (16.3% in obese NAFLD vs. 33.6% in obese controls; *p* = 0.004). These results may suggest that the main pathomechanism of metabolic disorders could be different in NAFLD patients in comparison to obese non-NAFLD subjects. In obese individuals with normal glucose tolerance, the volume of β-cells was found to be bigger than in controls [29], and it was shown that in order to maintain normal glucose tolerance, a compensatory hyperinsulinemia occurs which, in the long term, leads to an increase in insulin resistance [30]. The enhanced expression of SOCS prevents the development of pathological immune responses, which lead to both type 1 and type 2 diabetes [31]. However, the functional analysis of the SOCS1−1656G>A polymorphism revealed that this genetic variant does not influence the transcriptional activity of the gene in response to cytokines or other factors [19], but it affects the SOCS1-dependent signaling pathway by modifying SOCS1 binding to the domain for IRS2 on insulin receptors (IRs) in insulin-sensitive tissues [10]. Intriguingly, in our group of non-obese NAFLD patients, the GG homozygotes have HOMA-IR values in the normal range, which was two times lower than in the AA homozygotes (mean 1.8 ± 1.3 in GG vs. 3.6 ± 2.2 in AA). It seems that the presence of homozygous GG variants of the *SOCS1* gene may be protective from the presentation of insulin resistance, but only in the early stages of hepatic steatosis since this difference disappears in the group of obese NAFLD. Thus, our findings suggest that SOCS-dependent regulation of insulin signaling may additionally modify the progress of insulin resistance in NAFLD patients. It is in line with the report showing the association between the SOCS1−1656G variant and higher insulin sensitivity in young healthy Caucasians [19]. Interestingly, it was also suggested that this *SOCS1* variant that affects insulin sensitivity early in life may add to the risk of obesity/higher BMI later in life [19].

Although NAFLD is generally associated with obesity, it can also develop in lean subjects. This subphenotype of NAFLD patients has milder features of metabolic syndrome when compared with obese patients, but in comparison to healthy controls, they have a higher prevalence of dyslipidemia and insulin resistance [32]. The association between insulin resistance and NAFLD is generally accepted [33]. HOMA-IR was suggested as a good screening test for nonalcoholic fatty liver disease, and its rates of 2.0 or more have enhanced diagnostic value in distinguishing nonalcoholic fatty liver disease carriers from control individuals [34,35]. Both metabolic syndrome (MetS) and NAFLD are associated with the interactions of adipokines, cytokines, inflammatory factors, and insulin resistance [36]. The suggestion that insulin resistance and central (visceral) adiposity are important factors in NASH, rather than total adiposity alone, was further strengthened by the presence of insulin resistance in the group of lean NASH patients [37]. In this study, we demonstrated that the maintenance of insulin sensitivity in overweight NAFLD patients was associated with the SOCS−1656G variant. This intriguing observation draws attention to the role of the *SOCS1* polymorphism as a new genetic factor affecting insulin sensitivity in NAFLD. However, in the long term, environmental factors such as unhealthy diet and lack of efficient exercise may override the protective effects of this genetic variant of the *SOCS1* gene.

Another interesting finding of this study is the observation that TG level may be a feature that distinguishes GG homozygotes from AA homozygotes as the mean value of TG levels was significantly lower in GG homozygotes in comparison to AA homozygotes, even among obese patients with NAFLD. It was reported that triglycerides and insulin resistance are independently associated with NAFLD in normal weight individuals [38,39]. However, our study showed that there was a group of NAFLD patients, specifically the carriers of GG homozygotes, who had both lower TG levels, irrespective of BMI, and delayed presentation of insulin resistance even if they were overweight.

Certainly, our findings are limited by the inherent limitations. The lack of ultrasound examination with assessment of liver steatosis with Hamaguchi score is an important one. The lack of data on lipid profiles or HOMA-IR in controls is an additional notifiable weakness, related to the fact that these tests are not routinely conducted in blood donors. Finally, the study would benefit and perhaps findings would be strengthened if NAFLD sample size, in particular with regards to lean subjects, were larger.

The observation that a distinctive phenotype of NAFLD may be associated with a genetic variant of *SOCS1* justifies further investigation of the pathophysiology of fatty liver diseases. The pathophysiological mechanisms implicated in NAFLD are not fully understood; therefore, specific genetic associations should be under detailed investigation, and one intriguing/promising candidate is the *SOCS1* gene.

## Figures and Tables

**Table 1:** Demographic data on the analyzed subjects.

Variables	NAFLD		Controls	
	Male (*n* = 86)	Female (*n* = 52)	Male (*n* = 500)	Female (*n* = 500)
**Age (years)**	44.5 (19–74)	53.5 (18–72)	26 (18–62)	22.5 (18–66)
**Weight**	100.4 (69–149)	81.6 (55–127)	81 (50–150)	62.5 (45–110)
**Height**	176.3 (162–194)	158 (142–171)	180 (157–200)	167 (147–190)
**BMI (kg/m^2^)**	31.8 (23–46)	32.5 (22–48)	24.9 (17–45)	22.7 (16–40)
**Hamaguchi score**	3 (2–4)	3 (2–4)	N.D.	N.D.
**AST, IU/1 (normal: <35)**	31 (12–171)	24 (13–125)	21 (12–35)	18 (10–35)
**ALT, IU/l (normal: <35)**	52 (17–187)	34 (10–275)	25 (10–34)	21 (8–35)
**GGT, IU/l (normal: <35)**	47 (17–2690)	43.5 (10–507)	24 (9–35)	22 (8–35)
**HOMA-IR**	2.9 (0.4–86.6)	3.5 (0.4–23.8)	N.D.	N.D.
**Glucose (mg/dL) (normal: <99)**	102 (48–370)	100.5 (83–282)	80 (70–99)	82 (73–98)
**TG (mg/dL) (normal: <150)**	125 (45–547)	123.5 (27–190)	N.D.	N.D.
**Cholesterol (mg/dL**) **(normal: <190)**	189 (100–726)	201 (116–420)	N.D.	N.D.
**Waist circumference (cm)**	106.8 (89–142)	101 (74–146)	89 (64–145)	77 (57–120)
**Hip circumference (cm**)	109 (94–134)	111 (89–145)	103 (77–155)	99 (78–138)
**WHR**	0.9 (0.8–1.2)	0.9 (0.8–1)	0.9 (0.7–1.1)	0.8 (0.6–1)
**Fat (kg)**	35.1 (18–60)	44.9 (30–56)	26.7 (8–64)	19 (5–52)
**Tissue activity (kg)**	64 (42–89)	46.3 (35–62)	54.6 (39–85)	43.8 (31–62)

NAFLD: nonalcoholic fatty liver disease; BMI: body mass index; HOMA-IR: homeostasis model assessment of insulin resistance; AST: aspartate aminotransferase; ALT: alanine aminotransferase; GGT: gamma-glutamyl transferase; TG: triglycerides; WHR: waist–hip ratio. N.D. not done. Continuous data are presented as median and range.

**Table 2:** Distribution of the SOCS1−1656G>A variant in the entire cohort of NAFLD patients and controls.

Frequencies	NAFLD (*n* = 138)	Controls (*n* = 1000)	OR (95% CI)	*p*	*x* ^2^
**Genotype**					
GG	38 (27.5%)	238 (23.8%)	1.2 (0.9–1.8)	0.3	0.9
GA	73 (52.9%)	494 (49.4%)	1.1 (0.8–1.6)	0.4	0.6
AA	27 (19.6%)	268 (26.8%)	0.6 (0.4–1.1)	0.07	3.3
**Allele**					
G/A	149 (54.8%)/127 (45.2%)	970 (48.5%)/1030 (51.5%)	1.0 (0.8–1.3)	0.08	2.9

**Table 3:** Distribution of the SOCS1−1656G>A variant for individuals characterized by BMI ≥ 30 kg/m^2.^

Frequencies	NAFLD BMI ≥ 30 (kg/m^2^) (*n* = 98)	Controls BMI ≥ 30 (kg/m^2^) (*n* = 104)	OR (95% CI)	*p*	*x^2^*
**Genotype**					
GG	28 (28.6%)	21 (20.2%)	1.6 (0.8–3.0)	0.2	1.9
GA	54 (55.1%)	48 (46.2%)	1.4 (0.8–2.5)	0.2	1.6
AA	16 (16.3%)	35 (33.6%)	0.4 (0.2–0.7)	**0.004**	8.0
**Allele**					
G/A	110 (56.1%)/86 (43.9%)	90 (43.3%)/118 (56.7%)	1.6 (1.1–2.5)	**0.009**	6.7

**Table 4:** Distribution of the SOCS1−1656G>A variant in healthy controls as well as association tests.

	Normal weight	Overweight			Obese		
Frequencies	BMI = 18.5–24.9 (kg/m^2^) (*n* = 610)	BMI = 25–29.9 (kg/m^2^) (*n* = 270)	OR (95% CI)	*p vs. normal weight*	BMI ≥ 30 (kg/m^2^) (*n* = 104)	OR (95% CI)	*p vs. normal weight*
**Genotype**							
GG	142 (23.3%)	68 (25.2%)	1.1 (0.8–1.5)	0.5	21 (20.2%)	0.8 (0.5–1.4)	0.5
GA	317 (52%)	126 (46.7%)	0.8 (0.6–1.1)	0.1	48 (46.2%)	0.8 (0.5–1.2)	0.3
AA	151 (24.7%)	76 (28.1%)	1.2 (0.9–1.6)	0.3	35 (33.6%)	1.5 (0.9–2.4)	0.03
**Allele**							
G/A	601 (49.3%)/ 619 (50.7%)	262 (48.5%)/278 (51.5%)	1.0 (0.8–1.3)	0.8	90 (43.3%)/118 (56.7%)	0.8 (0.6–1.0)	0.09

**Table 5:** Association between the SOCS1−1656G>A polymorphism and the clinical characteristics of the entire NAFLD cohort.

Variables	*SOCS1*			*p–value* GG vs. AA
	GG	GA	AA	
***N* (male/female)**	38 (23/15)	73 (48/25)	27 (15/12)	*NS*
**Age (years)**	46 (18–63)	48 (19–74)	50 (26–62)	*NS*
**BMI (kg/m^2^)**	32.2 (23.5–48.1)	32.0 (22.5–42.5)	30.6 (24.2–46.5)	*NS*
**Hamaguchi score**	3 (2–4)	3 (2–4)	2 (2–4)	*NS*
**AST, IU/l (normal: <35)**	30 (13–171)	28 (14–125)	32 (12–85)	*NS*
**ALT, IU/l (normal: <35)**	51 (10–166)	36 (12–275)	50 (13–187)	*NS*
**GGT, IU/l (normal: <35)**	46 (10–2690)	43 (11–507)	55 (16–193)	*NS*
**HOMA-IR**	3.2 (0.4–19.6)	3.0 (0.4–86.6)	3.3 (0.4–23.5)	*NS*
**Glucose (mg/dL) (normal: <99)**	102 (84–232)	102 (48–370)	101 (83–247)	*NS*
**TG** **(mg/dL)** **(normal: <150)**	115 (27–299)	122 (44–5473)	148 (45–472)	*NS*
**Cholesterol (mg/dL**) **(normal: <190)**	198 (116–299)	196 (100–726)	189 (132–394)	*NS*

BMI: body mass index, HOMA-IR: homeostasis model assessment of insulin resistance, AST: aspartate aminotransferase; ALT: alanine aminotransferase; ALP: alkaline phosphatase; GGT: gamma-glutamyl transferase; TG: triglycerides, NS: non-significant. Continuous data are presented as median and range.

**Table 6:** Association between the SOCS1−1656G>A polymorphism and the clinical characteristics of obese and overweight NAFLD patients.

Variables	Obese NAFLD BMI ≥30 (kg/m^2^)			Overweight NAFLD BMI = 25–29.9 (kg/m^2^)		
	GG	AA	*P–value GG vs. AA*	GG	AA	*p–value GG vs. AA*
***N* (male/female)**	28 (15/13)	16 (8/8)	*NS*	10 (8/2)	11 (7/4)	*NS*
**Age (years)**	46 (18–63)	49 (26–62)	*NS*	47 (26–60)	50 (35–60)	*NS*
**BMI (kg/m^2^**)	33.4 (30–48)	35.2 (30–46)	*NS*	27.4 (23–29)	29.2 (24–29)	*0.06*
**Hamaguchi score**	3 (2–4)	2 (2–4)	*NS*	3 (2–3)	2 (2–3)	*NS*
**AST, IU/l (normal: <35)**	31 (13–171)	34 (12–85)	*NS*	26 (16–43)	27 (14–75)	*NS*
**ALT, IU/l (normal: <35)**	51 (10–166)	53 (17–170)	*NS*	54.5 (17–87)	41 (13–187)	*0.09*
**GGT, IU/l (normal: <35)**	47 (14–2690)	57 (16–193)	*NS*	39 (10–93)	47 (20–190)	*0.05*
**HOMA-IR**	3.5 (0.4–19.6)	3.2 (0.4–23.5)	*NS*	2.1 (0.4–3.7)	3.3 (0.6–9.5)	***0.03***
**Glucose (mg/dL) (normal: <99)**	105 (87–232)	98 (83–247)	*NS*	98 (84–108)	106 (87–129)	*NS*
**TG (mg/dL) (normal: <150)**	133 (43–299)	161 (111–422)	***0.02***	98 (27–216)	127 (45–472)	*NS*
**Cholesterol (mg/dL) (normal: <190)**	199 (128–299)	188.5 (132–394)	*NS*	185 (116–254)	193 (142–267)	*NS*

BMI: body mass index, HOMA-IR: homeostasis model assessment of insulin resistance, AST: aspartate aminotransferase; ALT: alanine aminotransferase; ALP: alkaline phosphatase; GGT: gamma-glutamyl transferase; TG: triglycerides, NS: non-significant. Continuous data are presented as median and range.
